# Increased cathepsin D protein expression is a biomarker for osteosarcomas, pulmonary metastases and other bone malignancies

**DOI:** 10.18632/oncotarget.4140

**Published:** 2015-05-14

**Authors:** Timo Gemoll, Franziska Epping, Lisa Heinrich, Britta Fritzsche, Uwe J. Roblick, Silke Szymczak, Sonja Hartwig, Reinhard Depping, Hans-Peter Bruch, Christoph Thorns, Stefan Lehr, Andreas Paech, Jens K. Habermann

**Affiliations:** ^1^ Section for Translational Surgical Oncology and Biobanking, Department of Surgery, University of Lübeck and University Medical Center Schleswig-Holstein, Lübeck, Germany; ^2^ Institute of Medical Informatics and Statistics, University Medical Center Schleswig-Holstein, Kiel, Germany; ^3^ Institute of Clinical Biochemistry and Pathobiochemistry, German Diabetes Center (DZD), Düsseldorf, Germany; ^4^ Institute for Physiology, University of Lübeck, Lübeck, Germany; ^5^ Institute of Pathology, University Medical Center Schleswig-Holstein, Lübeck, Germany; ^6^ Department of Orthopaedics and Traumatology, University Medical Center Schleswig Holstein, Lübeck, Germany

**Keywords:** osteosarcoma, bone malignancies, two-dimensional gel electrophoresis, CTSD, mass spectrometry

## Abstract

Cancer proteomics provide a powerful approach to identify biomarkers for personalized medicine. Particularly, biomarkers for early detection, prognosis and therapeutic intervention of bone cancers, especially osteosarcomas, are missing. Initially, we compared two-dimensional gel electrophoresis (2-DE)-based protein expression pattern between cell lines of fetal osteoblasts, osteosarcoma and pulmonary metastasis derived from osteosarcoma. Two independent statistical analyses by means of PDQuest^®^ and SameSpot^®^ software revealed a common set of 34 differentially expressed protein spots (*p* < 0.05). 17 Proteins were identified by mass spectrometry and subjected to Ingenuity Pathway Analysis resulting in one high-ranked network associated with *Gene Expression, Cell Death and Cell-To-Cell Signaling and Interaction.* Ran/TC4-binding protein (RANBP1) and Cathepsin D (CTSD) were further validated by Western Blot in cell lines while the latter one showed higher expression differences also in cytospins and in clinical samples using tissue microarrays comprising osteosarcomas, metastases, other bone malignancies, and control tissues. The results show that protein expression patterns distinguish fetal osteoblasts from osteosarcomas, pulmonary metastases, and other bone diseases with relevant sensitivities between 55.56% and 100% at ≥87.50% specificity. Particularly, CTSD was validated in clinical material and could thus serve as a new biomarker for bone malignancies and potentially guide individualized treatment regimes.

## INTRODUCTION

Osteosarcomas are the most frequently diagnosed primary malignant bone tumors and mainly occur in young people [1, 2]. Despite efforts in new therapeutic modalities based on neoadjuvant chemotherapy followed by surgical resection and postoperative chemotherapy, overall survival rates rarely exceed 60-65% [3-5]. Further, a significant portion of osteosarcoma patients develop metastasis even after curative resection of the primary tumor. Unfortunately, these metastatic osteosarcomas often show resistance to chemotherapeutic agents that were initially effective for treatment of the primary tumor. Here, the 4-year disease-free survival rate is on average limited to only 6% [6]. Despite molecular and cytogenetic studies, causes of the development of osteosarcomas and metastases could not be sufficiently elucidated [7, 8]. The development of metastasis is a complex and multistage process involving local invasion, access to the circulation, seeding and eventual proliferation within a favorable distant organ [9-11]. Hence, opportunities to improve outcomes for patients who present metastases or are at risk for metastatic progression require a better understanding of tumor biology.

Against this background, studies of protein expression profiles (comparative proteomics) offer a great possibility to reveal the molecular background of human osteosarcoma and to detect potential biomarkers for new prognostic and therapeutic options. Two-dimensional gel electrophoresis (2-DE) combined with mass spectrometry (MS) has been applied in studies of various cancers, including colon, endometrium, and breast [12-14]. Alternative/complementary technologies, such as SILAC (stable isotope labeling by amino acids in cell culture), ICAT (isotope coded affinity tags), or protein arrays, have emerged recently, but there is up to now no technology that matches 2-DE in its ability for routine parallel expression profiling of large sets of complex protein mixtures. Although proteomic alterations associated with the pathogenesis of osteosarcomas have been investigated in a few studies [15-17], none of these proteomic approaches compared clinical material of osteosarcomas with pulmonary metastasis and other bone diseases offering novel, phenotype related insights potentially enabling individualized therapy [18, 19].

**Figure 1:**
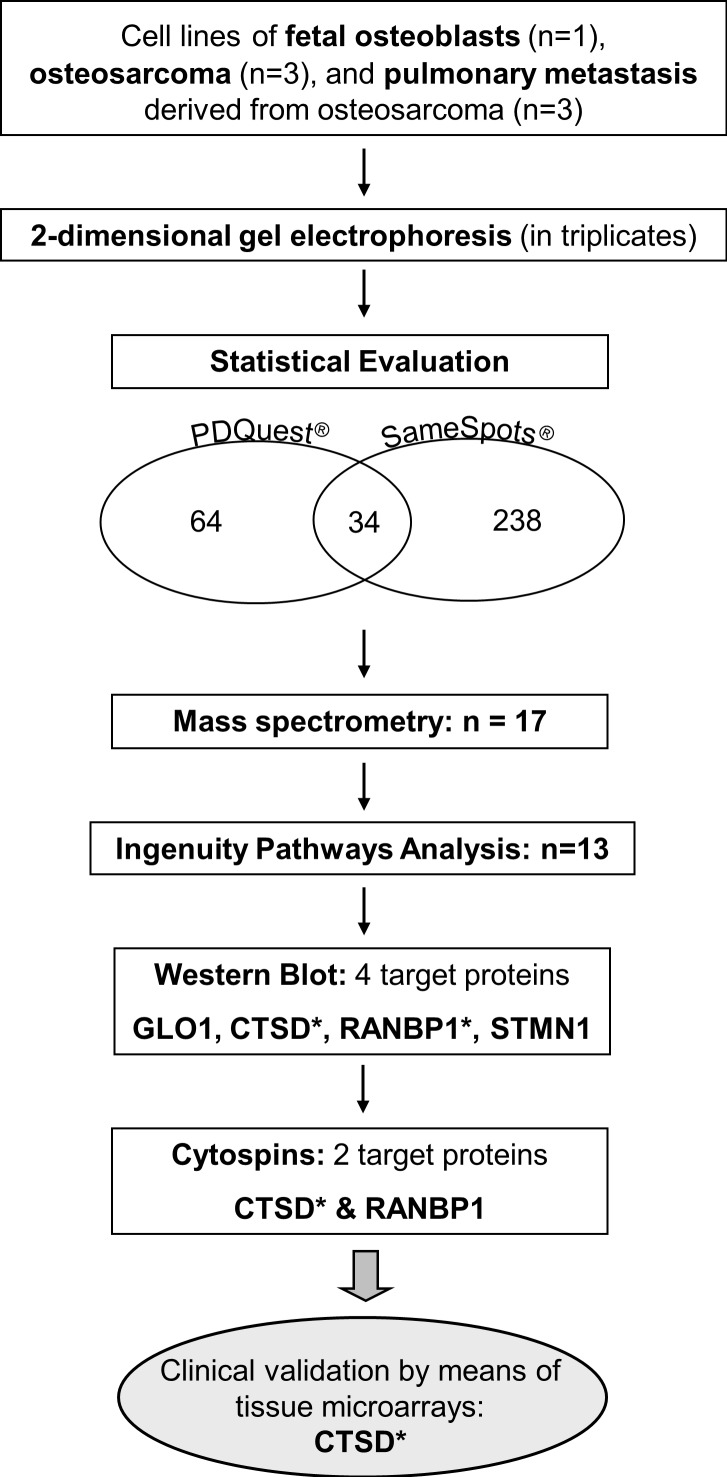
Workflow of the study design * target reached significance in individual validation steps.

In this study, our aim was to identify protein expression changes between fetal osteoblasts, osteosarcomas and pulmonary metastasis by means of 2-DE analysis, mass spectrometry, and pathway analysis. Identified candidate proteins were further validated using Western Blot and immunohistochemistry staining on cytospins and clinical tissues compiled on microarrays. For overall study design, please see Figure 1.

## RESULTS

### Expression profiles of fetal osteoblasts, osteosarcomas and pulmonary metastases

**Figure 2:**
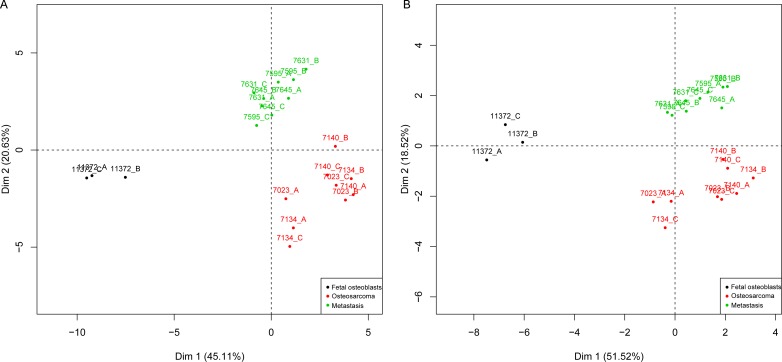
PCA map of the protein expression data Included are 34 significant (a) and 17 identified (b) proteins that were able to group fetal osteoblasts (black), osteosarcomas (red) and pulmonary metastases (green). X and y-axis show first and second principal components. Cell lines were run in triplicates **A.**, **B.**, **C.**

Two-dimensional gel electrophoresis (2-DE) was performed to detect proteins differentially expressed between fetal osteoblasts, osteosarcomas and pulmonary metastases derived from osteosarcoma. PDQuest and SameSpots software detected 1,791 and 1,114 spots per gel, respectively. In total, 238 differentially expressed spots (*p* < 0.05) between the three experimental groups were found, with 34 spots significantly detected in both, PDQuest and SameSpots analysis. For PDQuest analysis, only those spots were considered that were present in all gels (*n* = 1,108). All 34 spots were excised from the silver-stained gels, digested with trypsin, and analyzed by MALDI-TOF-MS followed by database search. In total, 17 spots could be identified (Table 1): 13 spots showed increased expression and four decreased expression in osteosarcoma and metastastic cell lines compared to the osteoblast cell line. Both, significant (*n* = 34) and identified (*n* = 17) protein spots showed a clear distinction of the groups in the PCA plots (Figure 2).

**Table 1:** Identified proteins of the differentially expressed protein spots

#	Protein Name	Symbol	Accession Number	Sequence Coverage(%)	Mascot Score	Regulation between groups*
1	Cathepsin D	CTSD	CTSD_HUMAN	28	122	↑
2	Ran-specific GTPase activating Protein	RANBPI	RANG_HUMAN	18	120	↓
3	Pre-mRNA-splicing factor SPF27	BCAS2	SPF27_HUMAN	11	87	↓
4	Ubiquitin carboxyl-terminal Hydrolase	UCHLI	UCHL1_HUMAN	36	115	↑
5	Putative high mobility group protein-like	LOC100130	HMGLX_HUMAN	21	51	↓
6	Lactoylglutathion Lyase	GLOI	LGUL_HUMAN	19	78	↑
7	Heat shock protein beta 6	HSPB6	HSPB6_HUMAN	21	90	↑
8	Stathmin OS	STMNI	STMN1_HUMAN	9	74	↓
9	Histone H4	HIST2H4A	H4_HUMAN	29	54	↑
10	Heat shock protein beta-1	HSPB1	HSBP1_HUMAN	48	155	↑
11	Cathepsin A	CTSA	PPGB_HUMAN	11	71	↑
12	Selenium-binding Protein	SELENBP1	SBP1_HUMAN	22	156	↑
13	Predicted:similar to hCG1654128	-	XP_001716101.1	7	62	↑
14	Ring finger protein 170	RNF170	RN170_HUMAN	10	50	↑
15	Actin-related protein 2/3 complex subunit	ARPC3	DQ328220.1	40	143	↑
16	Keratin Typ A	KRT1O	K1C10_HUMAN	16	107	↓
17	Ubiquitin carboxyl-terminal Hydrolase	UCHL1	AADO9172.1	52	172	↑

* ↑ **pro**tein is increased m the group of osteosarcoma and/or pulmonary metastasis compared to the group of fetal osteoblasts; ↓ protein is decreased in the group of osteosarcoma and/or pulmonary metastasis compared to the group of fetal osteoblast

### Pathway analysis (Effect of metastasis on biological networks)

Systems biology analysis by means of Ingenuity Pathway Analysis (IPA) was performed to estimate the impact of the identified proteins on biological networks and to select targets for downstream validation. Interestingly, IPA analysis detected just one network with a score of 36 containing 13 of the 17 differentially expressed proteins: ARPC3, BCAS2, CTSA, CTSD, GLO1, HIST1H4A, HSPB1, HSPB6, KRT10, RANBP1, SELENBP1, STMN1, and UCHL1 (Supplemental Figure 1). All proteins were associated with *Cell Death*, *Cancer* and *Hematological Disease* as high-ranked biological pathways and with *Cancer*, *Gastrointestinal Disease*, *Genetic Disorder*, *Cellular Assembly and Organization*, and *Cell Morphology* as top biological functions and disorders (0.0492 < *p* < 0.0001). V-myc myelocytomatosis viral oncogene homolog (MYC), stathmin1 (STMN1), cathepsin D (CTSD), and tumor protein p 53 (TP53) were central nodes of this network.

The identified proteins are involved in all aspects of tumor progression and metastasis. These proteins could be grouped into four functional Panther-database classes: (1) catalytic activity proteins (RANBP1, BCAS2, UCHL1, CTSA, CTSD), (2) structural molecule activity proteins (HSPB6, HSPB1, ARPC3, KRT10), (3) binding proteins (RANBP1, BCAS2), and (4) enzyme regulator activity proteins (RANBP1).

### Validation of protein expression by Western Blot and immunohistochemistry

Based on fold-change, availability of antibodies, pathway analysis and molecular function, four proteins were selected for downstream validation by Western Blot (GLO1, CTSD, RANBP1, and STMN1). Two proteins were increased (GLO1, CTSD) and the other two were decreased (RANBP1, STMN1) in osteosarcomas and metastases based on the 2-DE expression profile. Immunoblotting analysis proved that CTSD was over-expressed in sarcomas (*p* < 0.0083) and pulmonary metastases (*p* = 0.0061) compared to fetal osteoblasts, thus confirming the 2-DE data (Supplemental Figure 1). Likewise in agreement with 2-DE results, RANBP1 levels were increased in fetal osteoblasts compared to osteosarcomas (*p* = 0.0083) and pulmonary metastases (*p* = 0.0424) (Supplemental Figure 1). GLO1 and STMN1 showed a different regulation in the Western Blot as observed in the 2-DE analysis and were thus excluded from further downstream analysis.

**Figure 3:**
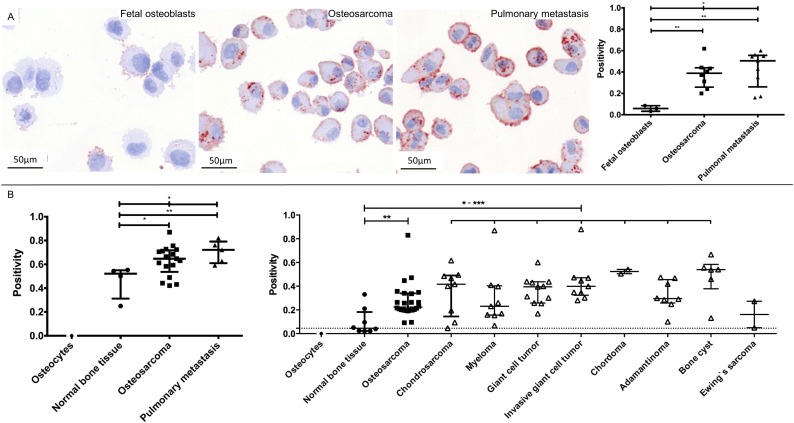
Representative images and data of the CTSD validation on cytospins of all cell lines (A) and tissue microarray validations (B) CTSD showed an increased staining from fetal osteoblasts, osteosarcomas to pulmonary metastasis (A, left) that reached significance between three groups (A, right). Customized (B, left) and commercially (B, right) tissue microarray-based evaluation of CTSD showed a strong overexpression in osteosarcomas, pulmonary metastasis and distinct bone diseases (except Ewing's sarcoma) compared to normal bone tissue. Based on the lack of representative fetal osteoblasts in the normal bone tissue, we chose isolated osteocytes – known to be descended from matured osteoblasts – as reference control (***: 0.0001 < *p* < 0.001; **: 0.001 < *p* < 0.01; * 0.01 < *p* < 0.05).

CTSD and RANBP1 were subsequently analyzed by immunohistochemistry on cytospins of the cell lines used. For CTSD, the median cytoplasmatic immunopositivity (IP) was 0.0586 in fetal osteoblasts, 0.3886 in osteosarcomas and 0.5046 in pulmonary metastases. CTSD expression reached significance between fetal osteoblasts and osteosarcomas (*p* = 0.0061), between fetal osteoblasts and pulmonary metastases (*p* = 0.0045) as well as between all three groups (*p* = 0.0127; Figure 3a). The median cytoplasmatic staining of RANBP1 showed no significance between groups and did not correlate with 2-DE and WB expression data.

For proof of clinical relevance, CTSD was chosen for additional immunohistochemistry validation on tissue microarrays (TMA). To confirm a higher expression of CTSD in pulmonary metastases compared to normal bone tissue and osteosarcomas, we build a customized TMA of 26 samples. In line with 2-DE and WB profiling, TMA-based immunohistochemistry revealed that CTSD was increased in osteosarcomas (*p* = 0.0237) and pulmonary metastases (*p* = 0.0079) compared to normal bone tissue and showed a significant regulation in the three group comparison (*p* = 0.0391). Hereby, CTSD reached a sensitivity of 76.47% at 100% specificity as well as a sensitivity of 100% at 100% specificity to predict osteosarcomas and pulmonary metastasis, respectively. Remarkably, a subsequent evaluation of a commercially available TMA of different bone entities (*n* = 176 tissues) showed the clinical impact of CTSD: CTSD immunostaining was located in the cytoplasm and showed a strong overexpression in osteosarcomas compared to osteocytes (Figure 3b). In detail, CTSD showed 0.2234 immunopositivity in osteosarcomas compared to no staining in all of the detected osteocytes (*p* < 0.0001) and 0.0450 immunopositivity in normal bone tissue (*p* = 0.0010). Thus, CTSD reached a sensitivity of 58.33% at 87.50% specificity for detection of osteosarcomas. Furthermore, CTSD expression was significant higher in chondrosarcomas (IP = 0.4166; *p* = 0.0039; sensitivity of 66.67% at 100% specificity), myelomas (IP = 0.2305; *p* = 0.0076; sensitivity of 55.56% at 87.50% specificity), giant cell tumors (IP = 0.3946; *p* = 0.0004; sensitivity of 90.91% at 87.50% specificity), invasive giant cell tumors (IP = 0.3991; *p* = 0.0002; sensitivity of 100% at 87.50% specificity), chordomas (IP = 0.5234; *p* = 0.0022; sensitivity of 100% at 100% specificity), adamantinomas (IP = 0.2952; *p* = 0.0023; sensitivity of 87.50% at 87.50% specificity), and bone cysts (IP = 0.5392; *p* = 0.0013; sensitivity of 83.33% at 87.50% specificity), but not in Ewing's sarcomas (IP = 0.1613; *p* = 0.2667; Figure 3b) compared to normal bone tissue.

## DISCUSSION

Previous studies at the DNA and RNA level have identified various genes for osteosarcoma development [11, 20]. Alterations of genes and pathways including retinoblastoma protein (RB) and p53 have been well accepted as contributing to the genesis of osteosarcomas. However, the exact molecular pathogenesis still remains unclear, especially none of those identified genes have been translated into valid protein markers that could aid in early diagnosis and effective treatment [21]. Overall, pathomechanisms of osteosarcoma development and progression are not fully understood yet [18].

This study aimed at detecting proteins differentially expressed between fetal osteoblasts, osteosarcomas and pulmonary metastases with therapeutic and/or prognostic value in terms of individualized medicine. We used an approach of protein separation by 2-DE coupled with protein identification by matrix-assisted laser desorption/ionization time of flight (MALDI) MS analysis and database search and identified 17 proteins as differentially expressed between all three groups. Systems biology analysis revealed association of these proteins with *Cell Death*, *Cancer* and *Hematological Disease* pathways and classified them into catalytic activity, structural molecule activity, binding, and enzyme regulator activity. Four proteins were selected for Western Blot validation and differential expression for CTSD and RANBP1 (Supplemental Figure 1) could be confirmed.

RANBP1 is a cytosolic Ran/TC4 binding protein and interacts specifically with RAN complexed with GTP but not GDP. RANBP1 has been shown to cooperate with Ran in nuclear transport mechanisms [22, 23]. The involvement of RanBP1 in cell cycle progression has not been directly investigated, although studies with mutant proteins suggest that cell cycle control by Ran required the RanBP1-interacting domain [24]. Further, it could be shown that a differential expression of RANBP1 yields multipolar spindles causing errors in centrosome duplication or segregation to daughter cells which can lead to genomic imbalances [25, 26]. The here presented study detected a down-regulation of RANBP1 in osteosarcomas and pulmonary metastases cell lines by means of 2-DE protein profiling and Western blotting. Against this background, it is notable that a down-regulation sensitizes cancer cells to the chemotherapeutic agent taxol, e.g. Paclitaxel or Docetaxel, which has not been included in recent protocols for the treatment of osteosarcoma [27]. However, the expression profile of RANBP1 could not be confirmed immunohistochemically using cytospins.

In contrast to RANBP1, CTSD was increased on the protein level in the sarcoma and metastatic cell lines with its highest expression in the sarcoma cell lines. Subsequently, CTSD differential expression could be confirmed also by immunohistochemistry not only using the cytospins of cell lines but also testing clinical tissues comprised on TMAs (Figure 3). The role of CTSD in cancer has been postulated as promoting tumor growth directly by degrading and remodelling the basement membrane and the interstitial stroma surrounding the primary tumor. In one of the recent reports, CTSD was found to be highly expressed in cells from the primary tumor tissue in late stage colorectal cancer and showed significant correlation with subsequent distant metastasis and shorter cancer-specific survival [28]. Overexpression of CTSD induced malignancy in adjacent epithelium of prostate stromal cells [29] and increased the malignant phenotype and metastatic potency of transformed rat cell line cells [30]. Zeillinger et al indicated that quantification of cathepsin D in head and neck squamous cell carcinoma tissue was significantly higher than in normal tissue and that CTSD was independent from other pathohistologic markers [31]. In breast cancer, this protease is an independent marker of poor prognosis [32], shows an up-regulation of CTSD mRNA [33] and represents a tissue marker with an increased risk of metastasis [32, 34]. In this context, CTSD seems to play an essential role in the multiple step process of tumor progression, including the stimulation of cancer cell proliferation, growth of micrometastasis, and the inhibition of tumor apoptosis in several carcinomas [35]. However, this is the first report describing increased expression of CTSD in osteosarcomas, its pulmonary metastases and other bone disorders in general (Figure 3). Spreaficao et al. indicated CTSD up-regulation comparing cell lines of human mature osteoblasts and osteosarcomas (SaOS-2 cell line) but did not confirm these findings in clinical samples of osteosarcomas or other bone diseases [36]. Based on our results, CTSD could therefore serve as a tumor marker for malignancies and metastasis of the bone. Interestingly, it has been shown that expression levels of CTSD in peripheral blood are predictive of survival in patients with melanoma treated with tremelimumab [37, 38]. Thus, CTSD could potentially become a new therapeutic target for bone malignancies guiding individualized treatment regimes.

In summary, our clinical proteomics workflow identified CTSD as an over-expressed protein in osteosarcomas and pulmonary metastases and may thus serve as a new biomarker for individualized treatment regimes for patients with osteosarcomas, even at metastastic stage. Additionally, our data indicate the potential of CTSD as a target for bone malignancies in general, thus addressing a high number of affected patients.

## MATERIALS AND METHODS

### Cell culture and cytospin

Human cell lines of fetal osteoblasts (CRL 11372), osteosarcomas (CRL7023, CRL7134, CRL7140) and pulmonary metastases derived from osteosarcoma (CRL7585, CRL7631, CRL7645) were purchased from ATCC-Promochem^®^ and cultured according to the manufactures' recommendations (www.lgcstandards-atcc.org) to a confluence of 80%. Culture conditions for all cell lines were 37°C with 5% CO_2_. For cytospins, 100μL of cell suspension were applied for cytocentrifugation (Cytofuge 2; Shandon Inc., Pittsburgh, USA) at 700 rpm for 5 min. For each cell line, three to five cytospin slides per passage were prepared. All cytospins and protein extraction for 2-DE and WB were produced simultaneously. Immunohistological staining of the cytospins was automatically evaluated by means of Image Scope (v9.1, Aperio, CA, USA). Data for immunopositivity (IP) on the molecular markers were collected as continues variables ranging from 0 to 1.

### Two-dimensional gel electrophoresis

Protein samples from all cell lines were analysed by two-dimensional gel electrophoresis (2-DE). For each cell line three different cell passage numbers were used. 17cm long IPG strips (Bio-Rad Laboratories, Hercules, CA, USA) with a pH range of 4 – 7 and 12.5% polyacrylamide gels were used. Cell lysates were prepared in lysis buffer and protein concentration was routinely determined [12, 13]. Briefly, 75 μg of each sample was diluted in 300 μL rehydration buffer. Passive rehydration and IEF was performed in a Protean IEF cell (Bio-Rad, Hercules, CA, USA) at 20°C, reaching approximately 53 kVhs. Prior to loading on the second dimension, focused IPG strips were equilibrated for 2 × 15 min in a buffer (50 mM Tris-HCl, 6 M urea, 30% glycerol, 2% SDS) containing 2% DTT in the first and iodoacetamid (2.5%) in the second step to reduce S-S bonds and alkylate free thiols. The gels were run overnight at constant 100 V and 12 °C to reach 2 kVhs. Gels were stained with silver nitrate, scanned with a 16-bit grayscale flatbed scanner (Image Scanner III, GE Healthcare, UK), and analyzed with PDQuest software (Bio-Rad, Hercules, CA, USA, version 8.0.1) and SameSpots software (Nonlinear Dynamics, USA, version 4.0) for detection of significantly expressed spots.

### In-gel digestion and mass spectrometry

2-DE-gel spots were manually excised. For in-gel digestion gel pieces were destained [39] and washed alternating for 10 min each in digestion buffer (25 mM ammoniumbicarbonate) and digestion buffer containing 50% acetonitrile (1:1, v/v). Neat acetonitrile was added and removed to dehydrate the gel pieces. The dry gel pieces were rehydrated with 10μL of an ice cold solution of 3.5 ng/μl trypsin (sequencing grade, Promega) in digestion buffer. Proteins were digested at 37°C for 4 h. Peptides were extracted for 30 min with 10 μL of 0.1% TFA and directly applied to a MALDI pre-spotted AnchorChip target (Bruker Daltoniks, Germany) according to the manufacturer's instructions. Subsequently, samples were analyzed in a time-of-flight Ultraflex-TOF/TOF mass spectrometer (Bruker Daltoniks, Germany). Acquired mass spectra were calibrated and annotated using Compass 1.3 software (Bruker Daltoniks, Germany) generating xml formated peak-lists. Results from each individual spot were screened against a human sub-set of Swiss-Prot (Sprot_57.8) non-redundant database using Mascot search engine (Version 2.2, Matrix Science Ltd, UK) with the following settings: enzyme “trypsin”, species “human”, fixed modifications “carbamidomethyl”, optional modifications “methionine oxidation” and missed cleavages “1”. Mass tolerance was set to 50 ppm. Using these settings, a mascot score of greater than 70 was considered significant.

### Pathway analysis

All identified proteins were analyzed using Ingenuity Pathway Analysis (IPA) software (Ingenuity Systems, CA, USA). Each protein symbol was mapped to its own protein object in the Ingenuity Pathways Knowledge Database. Networks of these proteins were assigned a score based on their direct connectivity. The score reflected the number of focus proteins in the network and how relevant this network was to the original list of focus proteins. A network graph was shown to present the molecular relationship between individual proteins.

### Western blot

A total of 10 to 50 μg of new prepared protein samples of cell lysate of fetal osteoblasts, osteosarcoma and pulmonary metastasis derived from osteosarcoma was separated by electrophoresis on a 12% SDS polyacrylamide gel and blotted onto PVDF membranes (Merck Millipore, MA, USA). After blocking with 5% non-fat dry milk (Bio-Rad, Hercules, CA, USA) in PBS containing 0.05% Tween 20, the membranes were probed with primary antibodies for GLO1 (Abcam; 1:600), CTSD (Abcam; 1:1,000), RANBP1 (Abcam; 1:1,000), and STMN1 (Abnova; 1:250). After incubation of membranes with a goat anti-mouse secondary antibody (Santa Cruz Biotec), final visualization was carried out with the ECL kit (Clearity Western ECL substrate, Bio-Rad, CA, USA) on the ChemiDoc™ imager (Bio-Rad, CA, USA). Densitometric analysis was performed using the ImageQuant software (GE Healthcare). The highest peak of each band with normalization against β-Actin (ACTB) was exerted to evaluate protein expression.

### Tissue-microarray

To detect and validate the expression of CTSD in clinical tissue, we performed immunohistochemical staining. To predict the metastasis potential, tissues of four normal bone tissues and 17 osteosarcomas with five corresponding pulmonary metastases were implemented into an in-house compiled tissue microarray (Supplemental Table 1) [13]. Clinical material was collected adhering to guidelines of the local ethical review board (# 13-157). For tumor progression in different bone diseases, a commercially available tissue microarray (TMA; BO2081, US Biomax, Rockville, USA) was used. After staining, duplicate cores of osteosarcomas (*n* = 24), chondrosarcomas (*n* = 9), myelomas (*n* = 9), Ewing's sarcomas (*n* = 2), giant cell tumors (*n* = 11), invasive giant cell tumors (*n* = 9), chordomas (*n* = 2), adamantinomas (*n* = 8), bone cysts (*n* = 6), and adjacent normal tissues (*n* = 8) were evaluated (Supplemental Table 2). Based on the lack of representative fetal osteoblasts in the normal bone tissue, we chose isolated osteocytes – known to be descended from matured osteoblasts – as reference control.

All tissue sections were deparaffinized in xylene, rehydrated in a gradient alcohol series and microwaved in an oven in a citrate buffer (pH 6.0) for 15 minutes at 95°C for antigen retrieval. Subsequently, the tissue sections were incubated with primary antibody (CTSD, Abcam, 1:1,000) at room temperature overnight. The next day, the sections were washed with phosphate-buffered saline three times for three minutes each and then incubated with a biotinylated goat antimouse IgG for 30 minutes at room temperature. Color reaction test was performed using the avidin-biotin complex (ABC) and the sections were counterstained with hematoxylin, followed by dehydration and mounting. IP of CTSD was analyzed using an automated computer system with positive pixel count: A robotic microscope (Pannoramic DESK, 3D Histech, Hungary) scanned each slide from which histological representative regions were assessed quantitatively by Image Scope (v9.1, Aperio, USA). One senior pathologist (C.T.) reviewed all slides after H&E staining. Immunopositivity of the molecular markers were collected as continues variables ranging from 0 to 1.

### Statistical analysis

SameSpot was programmed to select significant spots by an analysis of variance (ANOVA) with *p* < 0.05. PDQuest protein expression data were pre-processed by log transformation and all proteins with at least one missing value were excluded. A mixed effect model was fitted to determine proteins with different expression in the three groups. In addition to the fixed effects group, time point and their interaction, the parameter “cell line” was modelled as random effect. Proteins with a p-value for group < 0.05, p-value > 0.05 for the interaction term, maximal effect of group larger than maximal effect of time point and a least one mean difference in groups ≥ 1 were selected for MS. Fold-change (FC) was calculated as 2^(mean of group 1 – mean of group 2). PCA using expression data of all identified proteins was used to demonstrate that these proteins were able to discriminate cell lines of different groups. The statistical software package R version 2.10.1 was used for all statistical analyses with R packages nime version 3.1-96 (mixed effect model) and FactoMineR version 1.14 (PCA). For analysis of cytospins, Western Blots, and tissue microarray data, Mann-Whitney U test were calculated with alternative hypotheses based on observed expression differences in 2-DE gel data. Duplicated TMA-cores per case were averaged.

## SUPPLEMENTARY MATERIAL FIGURE AND TABLES


