# Supplementary material for: Increased cathepsin D protein expression is a biomarker for osteosarcomas, pulmonary metastases and other bone malignancies
Source: Oncotarget. 2015 May 14;6(18):16517–26. doi: 10.18632/oncotarget.4140 (PMC4599286; doi:10.18632/oncotarget.4140)
Supplement: Supplementary file 1 [file oncotarget-06-16517-s001.pdf]

Increased cathepsin D protein expression is a biomarker for osteosarcomas, pulmonary metastases and other bone malignancies

Supplementary Material

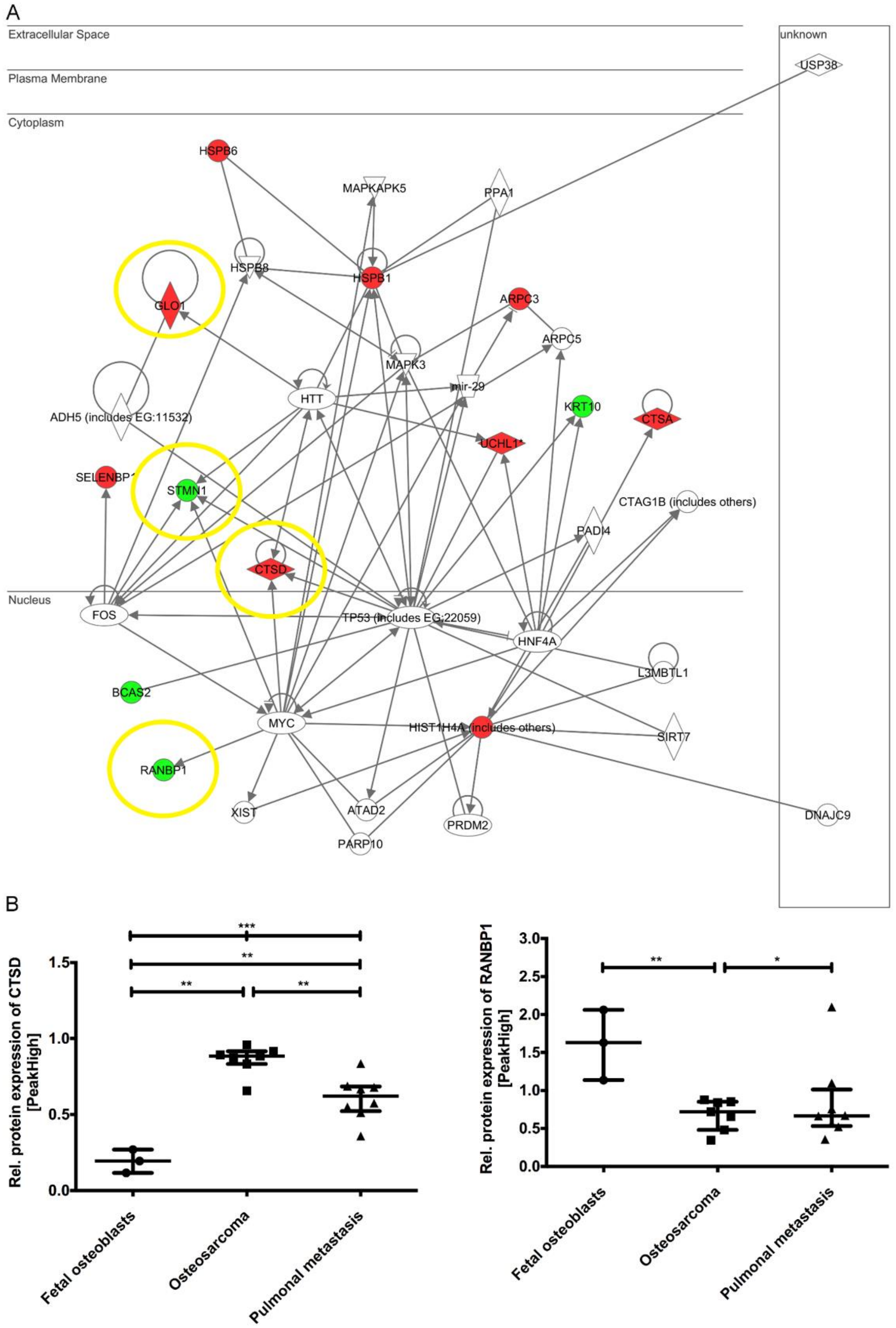

Supplemental figure 1: (A) IPA-based pathway analysis of differential expressed proteins. Proteins were colored by over- (red) and under-expression (green) between fetal osteoblasts, osteosarcoma and pulmonary metastasis cell lines. Yellow circles indicate targets

for validation by Western Blot. **(B) Western Blot analysis of validated target proteins CTSD (left) and RANBP1 (right).** CTSD is significantly lower expressed in fetal osteoblasts cell lines whereas RANBP1 is significantly higher expressed in fetal osteoblasts cell lines (***: $0.0001<p<0.001$; **: $0.001<p<0.01$; *: $0.01<p<0.05$). Plots show single protein expressions (peak high with normalization against ACTB) of each sample as well as median and interquartile range of each group.

Supplemental table 1: Evaluated patient cohort of the in-house compiled tissue microarray.

	Sex	Age [years]	Origin	Comment
**Normal bone tissues**				
Sample 1	male	39	Tibia	
Sample 2	male	68	Femur	
Sample 3	male	59	Tibia	
Sample 4	male	27	Femur	
**Osteosarcomas**				
Sample 1	male	14	Humerus	teleangiectatic
Sample 2	female	59	Femur	chondroblastic
Sample 3	male	39	Femur	fibroblastic
Sample 4	female	89	Femur	fibroblastic
Sample 5	male	35	Mandibula	chondroblastic
Sample 6	female	75	Tibia	giant cell-like
Sample 7	male	64	Mandibula	-
Sample 8	male	12	Tibia	osteoblastic
Sample 9	female	14	Fibula	teleangiectatic
Sample 10	male	38	Tibia	osteoblastic
Sample 11	male	67	Calvaria	osteoblastic
Sample 12	male	25	Tibia	osteoblastic
Sample 13	male	74	Mandibula	chondroblastic
Sample 14	male	27	Femur	osteoblastic
Sample 15	male	21	Femur	chondroblastic
Sample 16	male	21	Femur	chondroblastic
Sample 17	male	55	Tibia	-
**Pulmonary metastases***				
Sample 1	male	14	Inferior lobe	(teleangiectatic osteosarcoma in the humerus)
Sample 2	male	10	Inferior lobe	(chondroblastic osteosarcoma in the femur)
Sample 3	male	17	Superior lobe	(chondroblastic osteosarcoma in the femur)
Sample 4	female	12	Superior lobe	(osteosarcoma in the tibia)
Sample 5	female	41	Superior lobe	(osteoblastic osteosarcoma in the tibia)

* Primary tumor of the pulmonary metastases is presented in brackets

Supplemental Table 2: Evaluated patient cohort of the commercially available tissue microarray (TMA; BO2081, US Biomax, Rockville, USA)

	Sex [male/female]	Average age at diagnosis [years]	Range [years]
**Osteosarcomas**	15/9	24.9	10-69
**Chondrosarcomas**	7/2	31.2	13-47
**Myelomas**	7/2	53.8	39-69
**Ewing's sarcomas**	0/2	54.5	15-94
**Giant cell tumors**	3/8	36.2	17-60
**Invasice giant cell tumors**	6/3	36.0	20-50
**Chordomas**	1/1	47.0	44-50
**Adamantinomas**	5/3	42.3	11-70
**Bone cysts**	1/5	27.3	16-45
**Adjacent normal tissue**	6/2	60.9	41-75
